# Evaluation of body-mapping shirts design for activities in warm environments

**DOI:** 10.1186/2046-7648-4-S1-A134

**Published:** 2015-09-14

**Authors:** Li-Yen Lin, Simon Annaheim, Psikuta Agnieszka, Faming Wang, Li-Chu Wang, Robert Jou, Sheng-fu Chiu, René Rossi

**Affiliations:** 1 Department of Testing and Certification, Taiwan Textile Research Institute, New Taipei City, Taiwan; 2 Swiss Federal Laboratories for Materials Science and Technology, EMPA, St. Gallen, Switzerland; 3 Department of Business and Planning, Taiwan Textile Research Institute, New Taipei City, Taiwan

## Introduction

The aim of this study is to explore the fundamental knowledge of design high performance body mapping t-shirts(BMT) for warm environments. Several factors were discussed including physical properties of fabrics, fitness of the t-shirts and comparing body-mapping versus traditional non-body mapping t-shirts.

## Methods

Five textile materials, including modified hydrophilic nylon(No1), moisture management fabric(No2), porous structure mesh fabric(No3), highly wicking knitted fabric(No4), single jersey with cooling printing(No5) were selected for this study and also a commercial body-mapping shirt was chosen as the control sample. The physical properties of the five selected fabrics were determined, i.e., thermal insulation, evaporative resistance, thickness and moisture management properties. The heat and mass transport and thermo-physiological impact of the samples were assessed by using a sweating torso and a thermal human simulator (THS). The thermal parameters were predicted during a two-hour exposure in 20 °C and 28 °C at 6 METs. Two of the studied fabrics were chosen to design BMT by locating to different clothing parts. The effect of clothing fit on the local heat exchange of BMT was examined as well. The BMT were sewn made-to-measure in two different designs, tight fit and regular fit. The thermal and evaporative properties of BMT were evaluated by using the manikin. Two phases were applied, dry heat loss was obtained from the first phase and a sweat amount of 110 g.h^-1 ^was applied to obtain wet heat loss during the second phase, where heating power in the individual zones was recorded to maintain surface temperature of manikin at 34 °C.

## Results

The initial cooling rate(IC) of the five selected fabrics were in the range of 5 °C.h^-1 ^- 13 °C.h^-1^, and the sustained cooling(SC) were in the range of 0.5 °C.h^-1 ^- 1.3 °C.h^-1^. Dry and wet heat loss from the trunk of manikin for tight fitting shirt designs were both 15 % higher than the regular ones.

## Discussion

Fabric sample-No3 showed an increased IC, while SC was better for fabric sample-No1. Dry heat loss from the trunk of manikin revealed an obvious difference (P < 0.001) between regular and tight fitting shirt designs. The effect of fitting was confirmed in case of wet heat loss as well. Highest wet and evaporative heat loss and therefore best overall cooling was found for the body mapping shirt with fabric sample-No1 in the upper trunk (chest and shoulders) and fabric sample-No3 in the lower trunk (abdomen and lower back).

## Conclusion

Fabric sample-No1 showed the best performance in chest and shoulders with high contact area and low air gap thickness. In the abdomen section, a more air permeable fabric (sample-No3) provided better cooling effects. As probably more air is trapped in this section, the higher air permeability contributed to more air exchange and, thus, removal of heat. Of particular interest is the upper arm section. The measurements revealed the best effects for a tight fitting shirt in combination with a high air permeable fabric (sample-No3). We suggest wearing a tight fitting shirt during physical activity in hot and humid conditions.
